# Combining sentiment analysis and text mining with content analysis of farm vet interviews on mental wellbeing in livestock practice

**DOI:** 10.1371/journal.pone.0304090

**Published:** 2024-05-22

**Authors:** Andrew J. Duncan, Madeleine K. Henry, Kate Lamont

**Affiliations:** 1 Northern Faculty, Department of Veterinary and Animal Science, Centre for Epidemiology and Planetary Health, Scotland’s Rural College (SRUC), Inverness, United Kingdom; 2 UHI Inverness, University of the Highlands and Islands, 1 Inverness Campus, Inverness, United Kingdom; University of KwaZulu-Natal College of Health Sciences, SOUTH AFRICA

## Abstract

**Background:**

The aim of the How Farm Vets Cope project was to co-design, with farm veterinary surgeons, a set of web-based resources to help them and others deal with the different situations that they can face. As part of the wider project, participants were recruited for one-to-one semi-structured phone interviews. These interviews focused on elements of job satisfaction and how the participants coped during periods of poor mental wellbeing or with setbacks and failure.

**Methods:**

Transcripts of these interviews were analysed using both quantitative methods of sentiment analysis and text mining, including term frequency/inverse document frequency and rapid automated keyword extraction, and qualitative content analysis. The twin aims of the analysis were identifying the important themes discussed by the participants and comparing the results of the two methods to see what differences, if any, arose.

**Results:**

Analysis using the afinn and nrc sentiment lexicons identified emotional themes of anticipation and trust. Rapid automated keyword extraction highlighted issues around age of vets and support, whilst using term frequency/inverse document frequency allowed for individual themes, such as religion, not present across all responses, to be identified. Content analysis supported these findings, pinpointing examples of trust around relationships with farmers and more experienced vets, along with some examples of the difference good support networks can make, particularly to younger vets.

**Findings:**

This work has confirmed previous results in identifying the themes of trust, communication and support to be integral to the experience of practicing farm veterinary surgeons. Younger or less experienced vets recognised themselves as benefiting from further support and signposting, leading to a discussion around the preparation of veterinary students for entry into a farm animal vet practice. The two different approaches taken showed very good agreement in their results. The quantitative approaches can be scaled to allow a larger number of interviews to be utilised in studies whilst still allowing the important qualitative results to be identified

## 1. Introduction

Vets experience a range of challenges which can have a negative impact on their mental health and wellbeing [1,2]. Lovell and Lee [3] highlighted risks around burn out and compassion fatigue whilst Clarke and Knights [4] observed that vets have a tendency to turn unrealistic ideals of expertise back on themselves, which generates doubt and insecurity, resulting in them slipping into cycles of worry and self-doubt. They suggested that vets have problems, not just in persuading clients of their competence, but also in persuading themselves. A positive psychology-based approach, in which a veterinary career is depicted as a richly generative source of satisfaction and fulfilment [5], can be used to support protective factors against the impacts of ‘imposter syndrome’ and challenges related to the role.

As part of its annual renewal of membership, the Royal College of Veterinary Surgeons has been asking members to indicate the area in which they most commonly work. The most recent data shows that 1,675 gave their primary species type as "farm animal" and 1,686 responded with "mixed" compared with 15,902 who stated that it was small animal. (Pers. Comm.) It is therefore understandable that the majority of research on mental health and wellbeing within the profession would be focussed on vets in small animal practice. Although there will be similarities across the profession, given the differences in daily routine between farm and small animal veterinary surgery, the challenges experienced by vets working in these settings are likely to be different [6]. There is a need to understand the impact on farm vets of the risk factors described above.

By focussing on the positives, it is possible to build resilience in veterinary practice [7]. Therefore, a greater understanding of job satisfaction of a livestock vet is important in understanding resilience to the challenges of the role.

Wallace [8] suggested that work conditions which are emotionally exhausting for veterinarians may foster suicidal thoughts but that a supportive work environment is a valuable coping resource. Adam et al [9] stated that it was "highly likely that being called out to a farm at night and feeling unprepared to deal with the case and unable to access support will negatively affect young vets’ confidence and ultimately lead to them changing their career path".

The veterinary profession in the UK currently faces workforce challenges in relation to high levels of attrition and large numbers of vacancies [10,11]. These challenges are particularly acute in rural, large animal and mixed practices [12]. There is a need to avoid attrition due to emotional, ethical and moral struggles in order to sustain the future workforce.

Whilst text mining has been used to conduct a review of peer reviewed suicide research [13], it has not been applied so thoroughly in relation to veterinary mental health. Sentiment analysis has been used to show that the publication bias toward reporting significant results is more positively skewed in veterinary clinical trials than in human trials [14] whilst text mining, including keyword extraction, has demonstrated good results when combining or analysing veterinary patient records [15]. Text mining techniques have also been used to construct an electronic resource containing veterinary terminology that will allow automated monitoring of free-text veterinary reports [16], assist with monitoring trends and risk factors in equine antimicrobial prescriptions [17], and identify the main research topics within Mountain livestock farming [18].

Brscic et. al. [19] applied text analysis techniques, including topic modelling, to the scientific literature around themes of suicide, burnout and depression in both veterinarians and veterinary students. The themes they identified led them to advise that veterinary students should be better prepared for the emotional, ethical and moral struggles of their profession and that ongoing training should be used to help them cope.

In a recent review on the stresses and strains amongst veterinarians, Pohl et al [20] highlighted similar themes as Brscic et al [19]—the need for support within workplaces and organisations, along with improving an individual’s ability to cope with stress caused by their work environment. The How Farm Vets Cope project [21] set out to gather the views and experiences of farm animal vets to identify mechanisms which could be used (a) to promote job satisfaction and (b) to break the cycle of negative thoughts that occur during periods of poor mental wellbeing or in response to setbacks and failure. As part of this project, semi-structured interviews were conducted with volunteer participants. Herein we present the results from two separate analysis methods, sentiment analysis/text mining and content analysis, applied to transcripts of these interviews with the aim of identifying the important themes surrounding job satisfaction and wellbeing that arose, and to assess how the two approaches integrate.

## 2. Materials and methods

### 2.1 Background and participant recruitment

This work was completed as part of the How Farm Vets Cope project [21]. The aim of this project was to co-design, with farm veterinary surgeons, a set of web-based resources to help them and others deal with the different situations that they can face. As part of the wider project, participants were recruited for one-to-one phone interviews. This was done via the use of direct messages on Twitter, email and phone contact with veterinary surgeons who previously indicated a willingness to participate, and through ‘sign up’ fliers at the British Cattle Veterinary Association Congress in October 2019. In addition, some veterinary practices and groups of farm veterinary practices shared information about the project to their staff. The purpose of these interviews was mainly to inform later workshops and to provide quotes for use in the web-based resources.

Vets who expressed an interest in taking part were sent a participant information sheet and consent form by email. Two follow up emails were sent if no response was received. Participants were recruited in the period between September 20th, 2019 and February 28th, 2020. Following recruitment, semi-structured interviews with 32 vets were conducted by telephone and recorded. A completed Consolidated criteria for reporting qualitative research (COREQ) [22] checklist is included in the supplementary file *COREQ_Checklist*.*pdf*.

Participants were asked if there were routine occupational activities that positively or negatively impacted on their mental health and wellbeing, and about the strategies they used to cope with the challenges they faced. Due to the nature of the data being recorded and the recruitment process, the project team had access to information that could identify participants. This information did not form part of the analysis. A total of 85 individual expressions of interest were received and information about the project provided to each. 32 interviews were consented to, 31 were successfully arranged and 30 of these were transcribed for analysis. The interviews were not of a fixed length with most between 30 and 60 minutes. Three interviews were just under half an hour, whilst four lasted over one hour.

Vets were not asked about their current employment status or location, but many shared this information during their interviews. Recruitment was based in the UK and of the resulting participants, all but one were based in the UK; one vet was currently working abroad but had previously worked in the UK. Most were currently working in farm animal practices, some were in mixed practice, others had retired or were working for government agencies or industry.

The veterinary experience of participants was also not explicitly sought, but as above, some (*n* = 14) shared this information. One vet had only been qualified for 18 months whilst it was 42 years since the most experienced had graduated. The SRUC Social Science Ethics Committee approved the study on the 18^th^ September 2019.

### 2.2 Sentiment analysis and text mining

To enable sentiment and text analysis of these transcriptions, the interviews were imported into the statistical software R [23] using the officer [24] package and tidied into a corpus with the participant id (1 to 30) and question recorded. Several steps of data cleaning were carried out to ensure amongst others, consistent spelling and removal of plural terms. Common words were recorded as “stop words” and were removed for most of the analysis. The stop words removed were based on the SMART (System for the Mechanical Analysis and Retrieval of Text) Information Retrieval System [25,26] and the researchers added the stop words *"yeah"*, *"yeh"* and *"work"*. Following the data cleaning, sentiment analysis was carried out using two sentiment lexicons, afinn [27] and nrc [28].

The afinn lexicon gives a word an integer value between -5 and 5, where each word has been assigned their value by hand [27]. The value classifies the individual word as either positive (value between 1 and 5), negative (value between -1 and -5) or neither (value of 0 or no value given), with the increasing absolute values indicating greater positivity or negativity. If a word is not included in the lexicon, this word was removed from this step of the analysis. In total the afinn lexicon contains 2,477 distinct words. The number of times where a word’s sentiment value could be mis-scored, because it is preceded by a negation, was also analysed. The words considered to be a negation were “not”, “no”, “never”, “without”, “don’t”, “didn’t”, “wasn’t” and “doesn’t”. To consider the overall effect of any negation during an interview, the sentiment value of any negated word was multiplied by -1.

The second sentiment lexicon used, the nrc lexicon, evaluated the words used by the participants as positive or negative and/or being associated with one or more of the emotions of anger, anticipation, disgust, fear, joy, sadness, surprise and trust. In total, the nrc lexicon contains 6453 unique words and each word can be associated with multiple emotions. This lexicon was also put together by hand but used crowdsourcing to help with the classifications [28].

In addition to the sentiment analysis, Rapid Automatic Keyword Extraction (RAKE) [29] was used to extract bigram keywords (or keyword pairs) from the interviews and the term frequency/inverse document frequency (*tf-idf)* was calculated for each individual interview. The *tf-idf* of a word (or bigram) is the term frequency (number of times it appears in the document) multiplied by the inverse document frequency (Silge & Robinson, 2016). The final equation is shown in (1).


$$tf-idf(term)=term\;frequency\times \ln\left(\frac{num.of\;documents}{num.of\;documents\;containing\;term}\right)$$ (1)

The higher the *tf-idf*, the more often a particular interview contains that term (or bigram) in comparison to how often it is used across the other interviews. With this measure we would not expect to see common words, such as “good” or “bad”, appear as they are used by all participants.

All analysis was carried out using the stopwords, tidytext, tidyverse and udpipe packages [26,30–32]. Plots were produced by the ggplot2, ggwordcloud and treemapify packages [33–35].

### 2.3 Content analysis

In addition, and in isolation, to the sentiment analysis described above, content analysis was carried out on the interview transcripts using NVIVO software [36] by the senior (third) author. Contents were coded into nodes and these nodes were further grouped into a set of aggregated nodes. The aggregated nodes were used to aid understanding, particularly in comparison to the quantitative results [37]. The work completed in the content analysis by the senior author was reviewed by the second author, a fully qualified veterinarian with experience of livestock, to ensure the relevance of the aggregated nodes to farm veterinary surgeons. This approach was undertaken to understand the characteristics of the interviews, including who said what and to what extent their experiences influence their ability to cope [38,39]. For further details, please see the supplementary file *COREQ_Checklist*.*pdf*.

## 3. Results

### 3.1 Sentiment analysis and text mining

**Fig 1:**
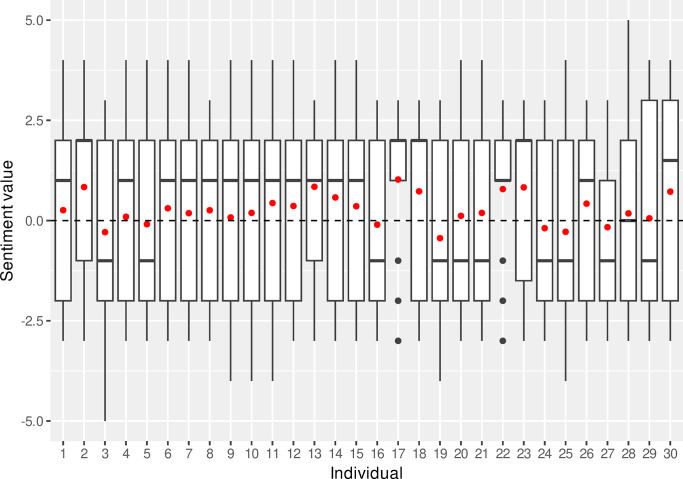
Overall sentiment score based on the afinn lexicon for each participant interview. Red points indicate the average value.

The first step in the quantitative analysis was to evaluate the words using the afinn sentiment lexicon. If we remove those words with no sentiment value, Fig 1 shows the range of sentiment for each interview with the red points indicating the average (mean) value. In total 7 participants had a negative mean sentiment value with the remaining 23 interviews having a positive mean sentiment score.

**Fig 2:**
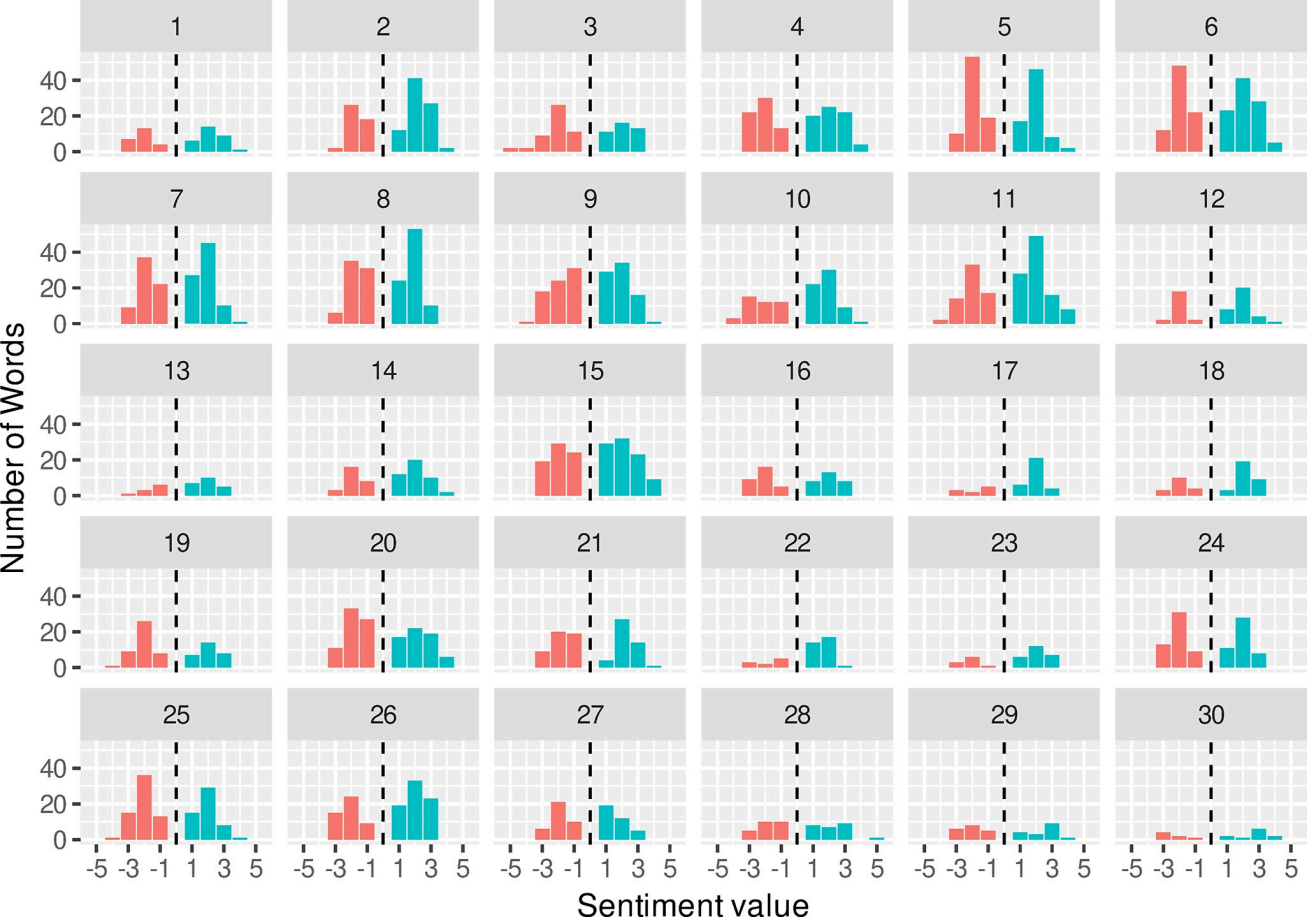
Percentage of words with sentiment values used during each participant interview. Words with a negative sentiment are shown in red, with positive in blue.

Fig 2 shows the total number of words classed as positive or negative via the afinn sentiment score and shows that participants had a reasonably even split over the course of their interview.

**Fig 3:**
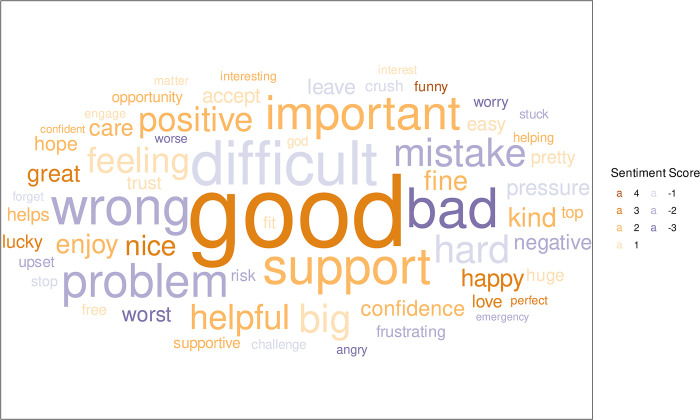
Word cloud for all participants, with the most popular words (appear at least ten times across interviews) coloured by their sentiment value and size proportional to the number of times they are used by the participants.

The overall popularity of particular words, with their sentiment scores, is shown in Fig 3. The words used by all participants that appear at least 10 times across all interviews are shown coloured by their sentiment value and with their size proportional to the number of times it is used. Whilst *“good”* and *“bad”* are clearly visible, *“mistake”* and *“support”* are also used many times.

**Fig 4:**
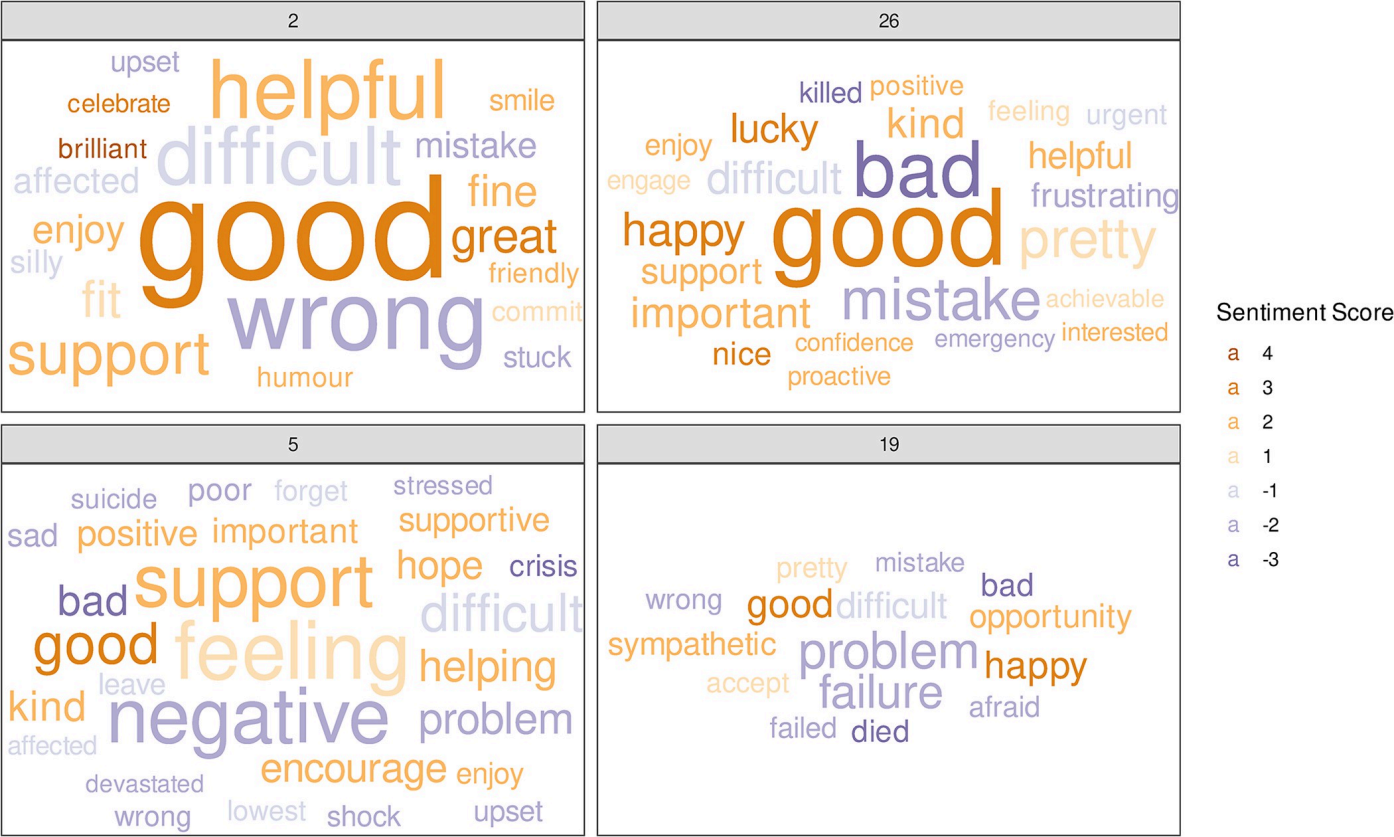
Word clouds for two participants with positive overall sentiment (2 and 26) compared to two participants classified as negative overall (5 and 19). Positive words are yellow/orange with negative words shown in shades of purple. The size of the word is proportional to the number of times it is used by each participant.

Selecting two participants (2 and 26) with an overall positive sentiment and comparing these with two with an overall negative sentiment (participants 5 and 19), Fig 4 shows the individual positive and negatively classed words used by each. Similarly to Fig 3, the words are coloured by their sentiment value and with their size proportional to the number of times it is used. These word clouds allow us to identify single words that are commonly used by individuals.

Fig 4 shows that participant 2 used the positively classified word *“helpful”* more than the others, with the positive word *“good”* used often by both positive participants. Of the two participants judged overall to be negative, participant 5 used *“negative”* the most, whilst participant 19 had fewer words classified and used *“problem”* or *“failure”* most frequently.

**Fig 5:**
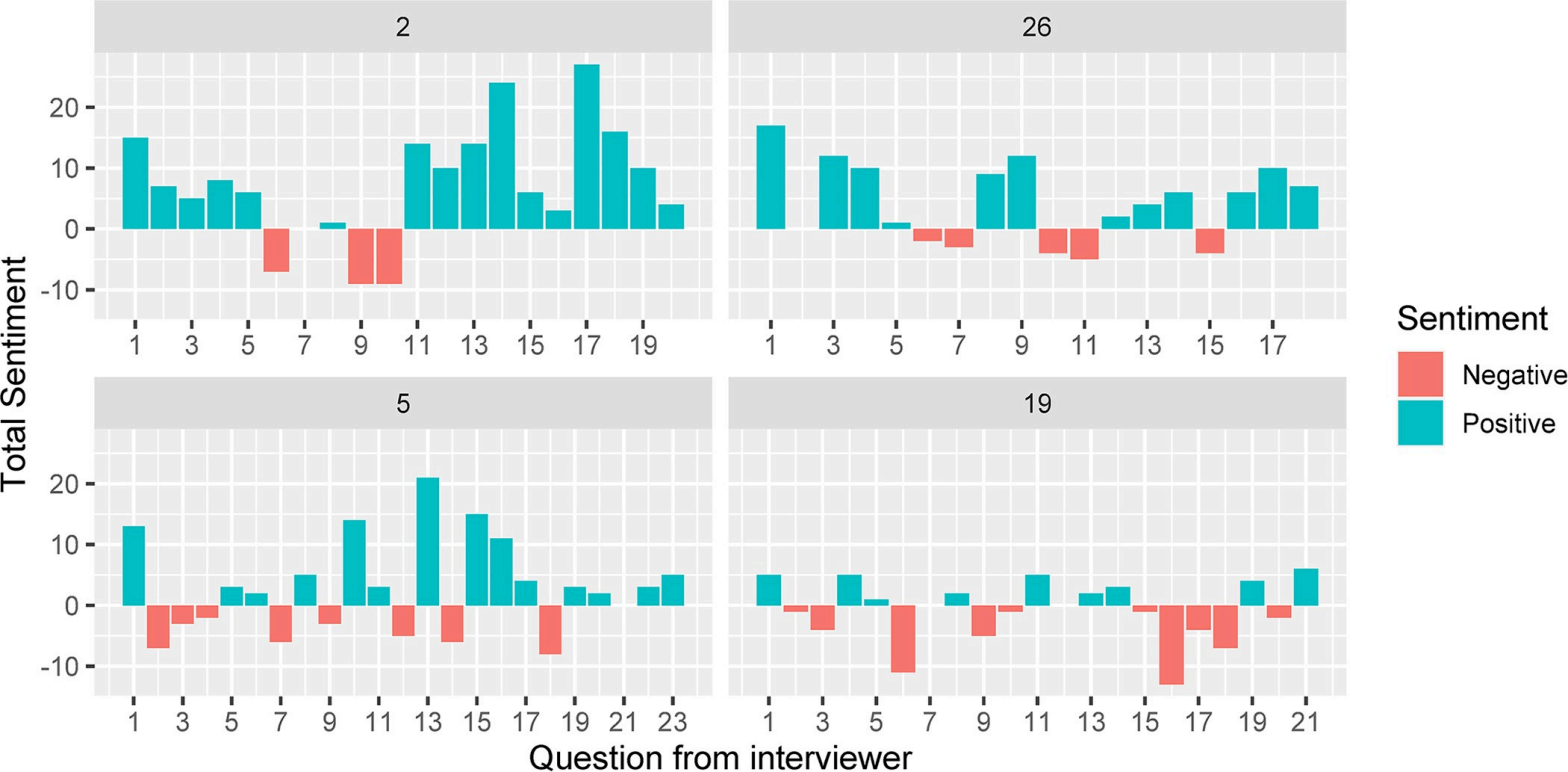
Total sentiment value, using the afinn sentiment score, for each question from selected participants over the course of the interview.

Within the interviews, the interviewer would either ask a direct question or provide a comment based on a previous response. As such it is expected that the sentiment would change over the course of the interview. Fig 5 shows the change in sentiment for the same four participants over time. Due to the semi-structured nature of the interviews and time available for each participant, the interviews varied in length, but the first question asked was “In your daily routine activities, what gives you job satisfaction?” and so we would expect a positive response. This was the case for all but three participants. The response from one of these participants was which scored a total of -1 due to *“Problems”* with a score of -2 and “*solved”* which has a score of 1. The follow up, or second question, asked the participant to identify challenges and it was expected to elicit a negative response. Fig 5 shows that this was not always the case and similar patterns were shown across the participants. Those participants selected for Fig 5 show that sentiment did vary throughout the interview and often changed from positive to negative as the interviewer picked up on specific parts of earlier responses. Due to the variation in length of interview, sentiment analysis over time is difficult to compare across all interviews.

*“Problems that can be solved quite quickly–e*.*g*. *calvings / lambings”*

**Fig 6:**
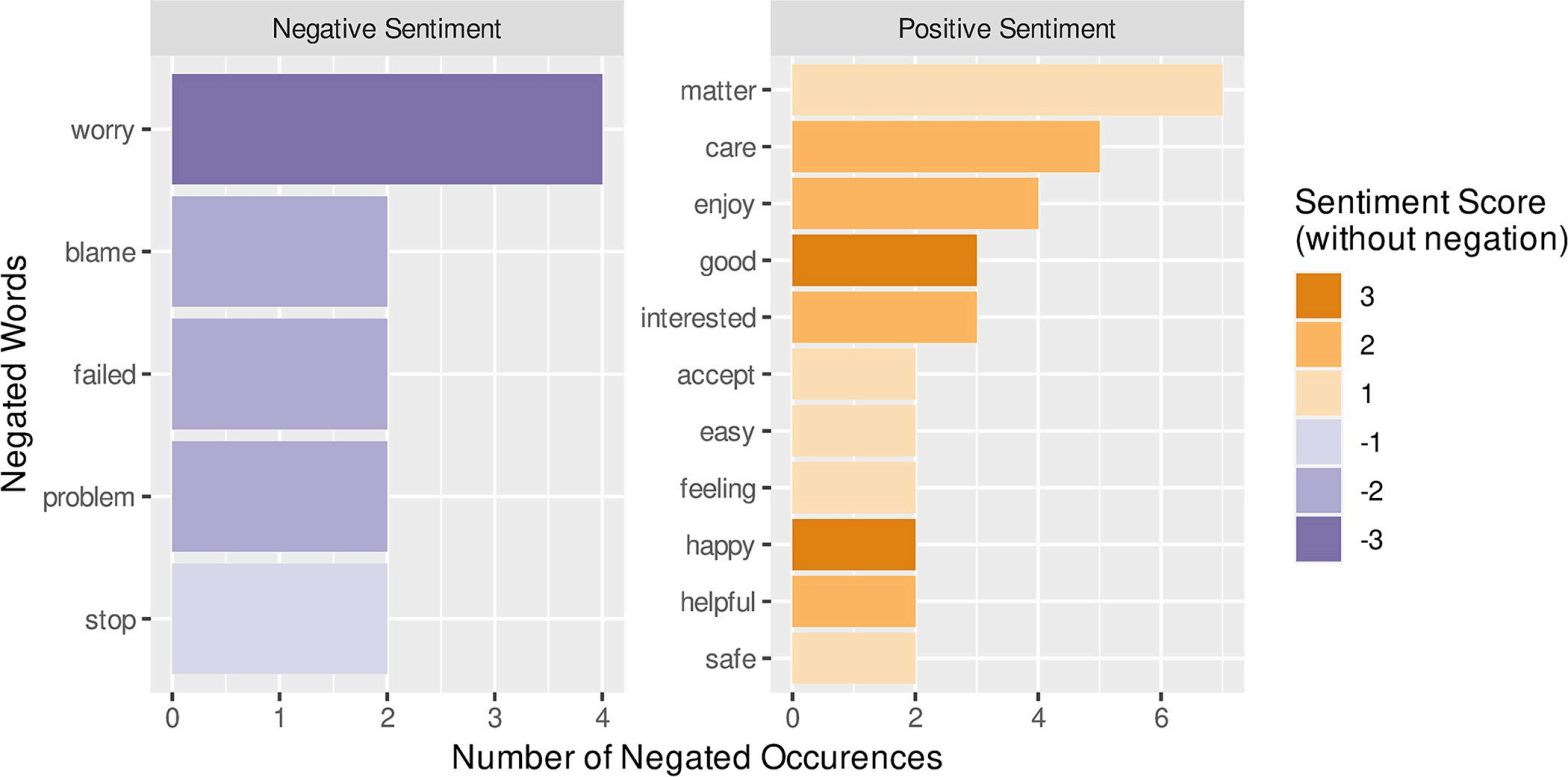
The words that are negated, more than once, during the interviews—order by the number of occurrences and coloured by their original (un-negated) sentiment score from the afinn lexicon.

Fig 6 shows that it was more common to negate a word with positive sentiment and the words *“matter”*, *“care”* and *“enjoy”* are negated more than any words with a negative sentiment score. Once the overall effect of negation had been accounted for, the mean sentiment scores of two participants changed from positive to negative (Fig 1). No participants changed their average sentiment score from negative to positive.

**Fig 7:**
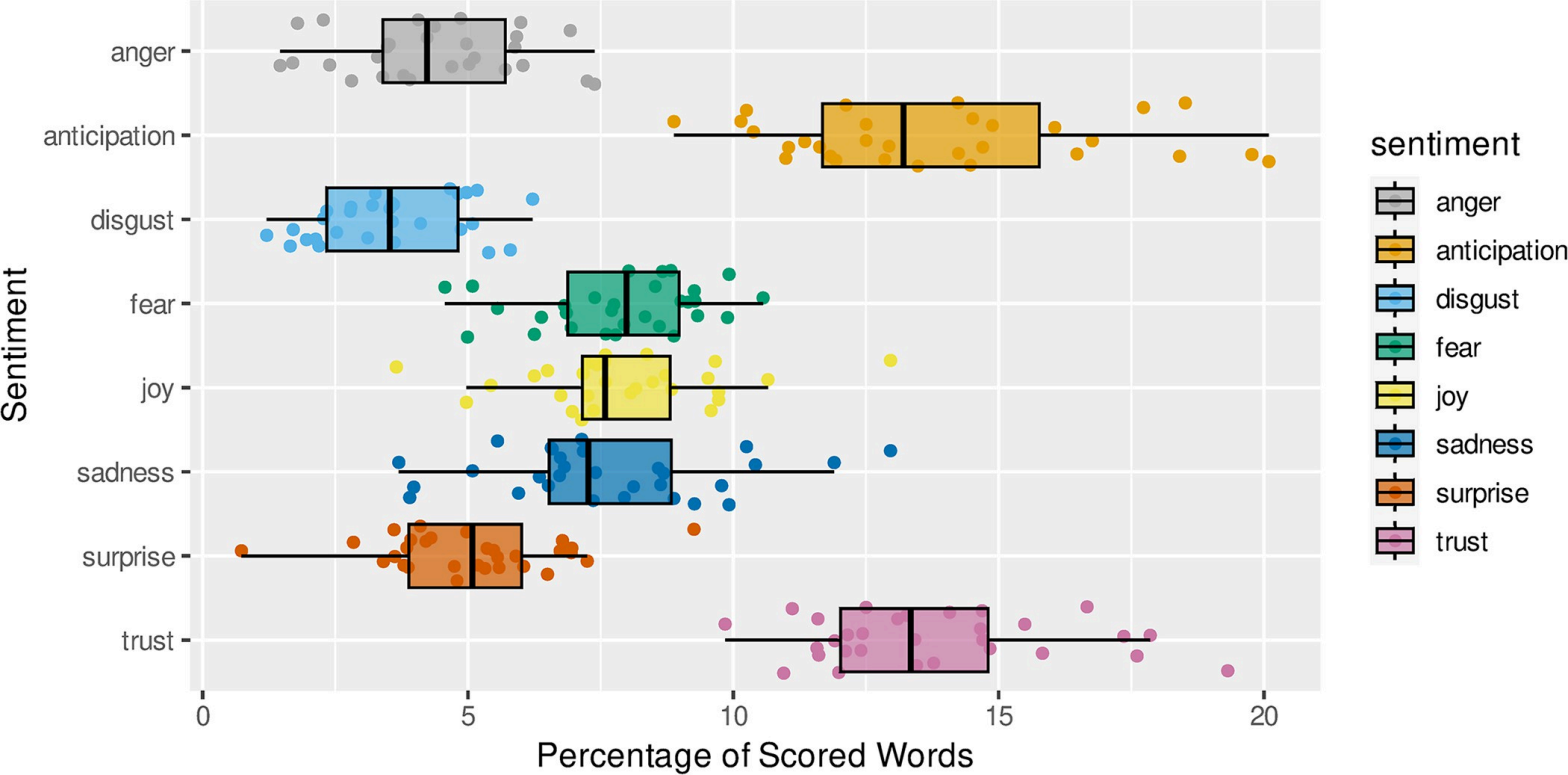
Percentage of scored words for all interviews classified with different emotional sentiment.

Fig 7 shows the percentage of words across all the interviews, categorised into different emotions using the nrc sentiment lexicon. The most common emotions, after the general positive and negative categories have been removed, are trust and anticipation.

**Fig 8:**
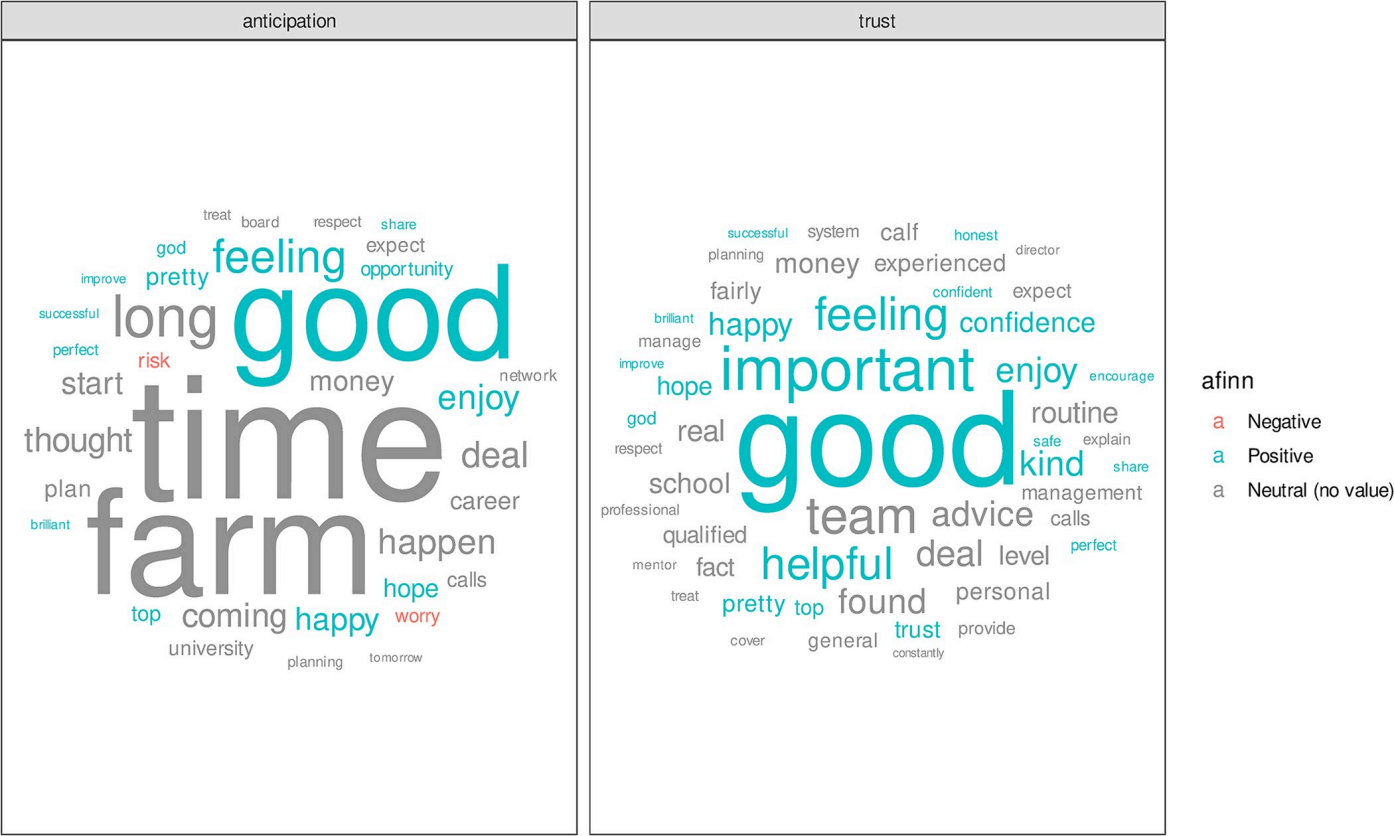
Most frequently used words across all interviews that were classed the emotional sentiment of Trust or Anticipation. Words are coloured by their afinn sentiment score, where words with no afinn value are labelled neutral. Only words which appeared at least eight times are included.

The nrc lexicon classifies words into one or more emotion and many of the emotions contain words that were classified by the afinn lexicon as either positive or negative. Fig 8 shows the most frequently used words classified with emotions of either Trust and/or Anticipation, combined with whether the word’s afinn sentiment score would be positive or negative. Whilst there are no words classified within the Trust emotion which also have a negative afinn score, Anticipation includes *“worry”* and *“risk”* which the afinn lexicon classifies as negative. The word “confidence” is also prominent in the Trust word cloud of Fig 8.

**Fig 9:**
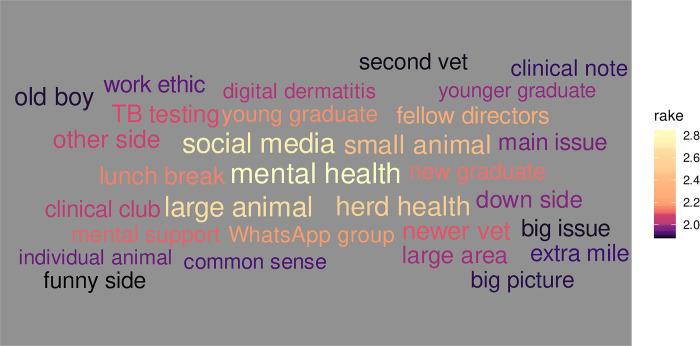
Keywords (bigrams) identified by top RAKE score across all interviews. The top 30 keyword combinations are shown, coloured by their RAKE value.

Thus far we have only considered sentiment based around individual words. The interviews were also examined in terms of bigrams (two-word groups) and trigrams (three-word groups). Fig 9 shows the keyword bigrams from all the interviews, based on RAKE score. We see high scores for the similar bigrams, in the context of the participants, of *"newer vet"*, *"young graduate"* and *"younger graduate"*.

**Fig 10:**
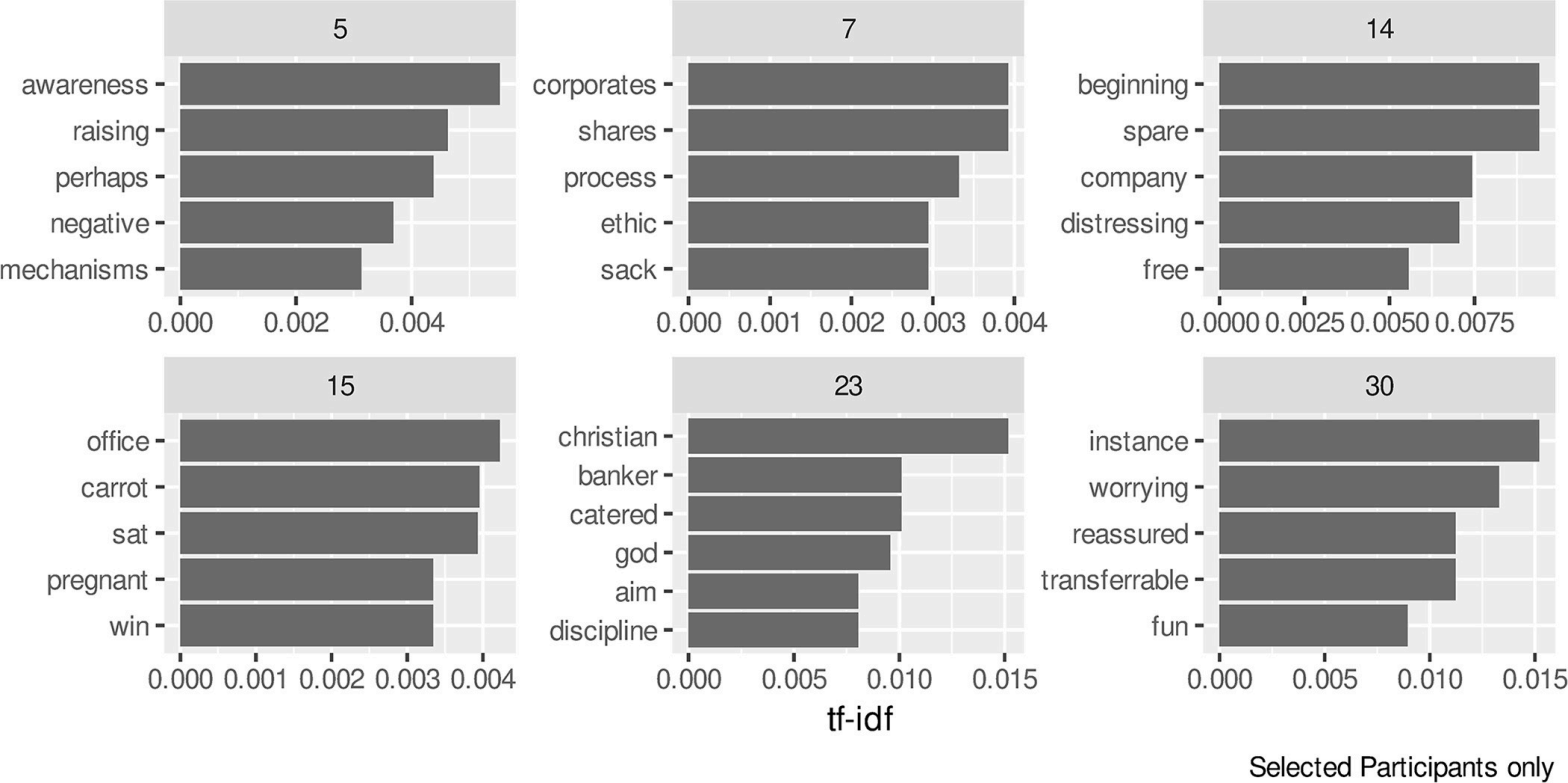
Individual words with the largest tf-idf values for selected participants.

**Fig 11:**
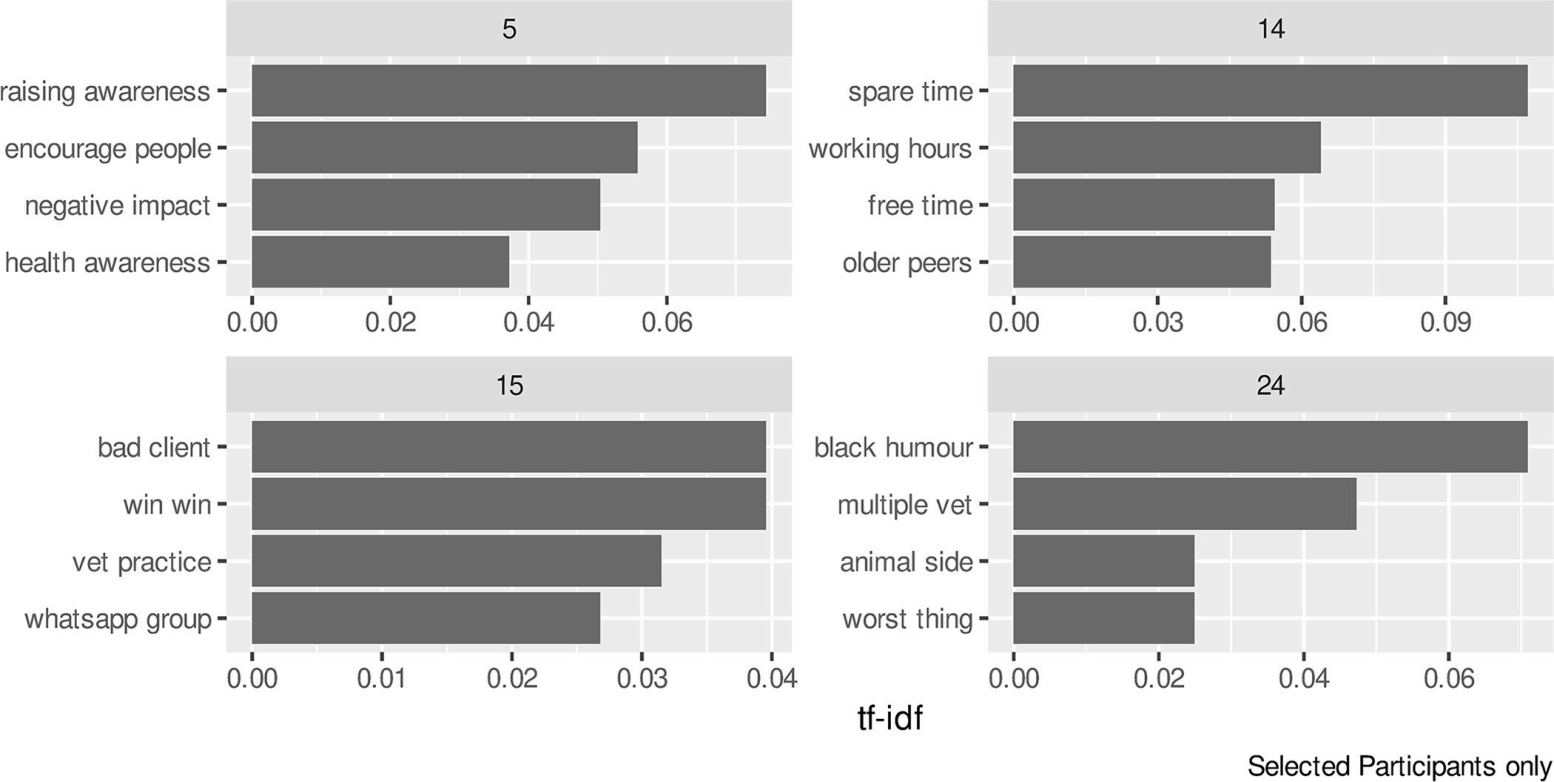
Bigrams with the largest tf-idf values for selected participants.

The final piece of text analysis carried out was to examine those words that might be particularly important within an individual interview. Whereas the RAKE score of Fig 9 shows the key bigrams for all the participants, Figs 10 and 11 show the individual words and bigrams respectively with the highest *tf-idf* values (1) for selected participants. The participants were selected to demonstrate a range of results shown with the *tf-idf* calculation. Several participants could not be shown as the words selected by the *tf-idf* could allow them to be identified.

The results in Fig 10 for participant 23 highlights religious themes within their interview. In Fig 11, participant 24 makes use of “black humour” which may indicate a particular coping mechanism whilst participant 14 refers to time across their results in both Figs 10 and 11, showing a possible theme of work/life balance within their interview. The bigram of *"Whatsapp group"* that appears in Fig 11 for participant 15 also appears with a high RAKE score in Fig 9 for the group as a whole.

### 3.2 Content analysis

**Fig 12:**
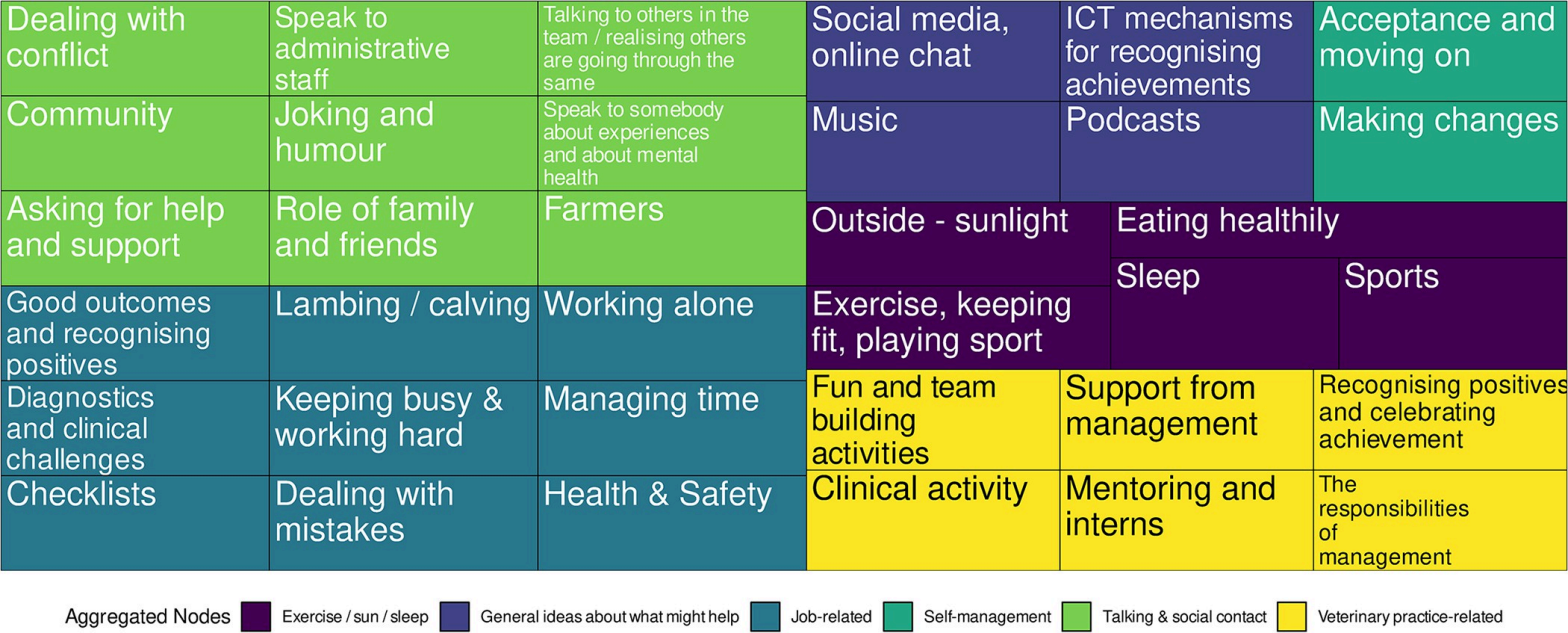
Treemap of the individual and aggregated nodes from the content analysis of the 30 interview transcripts.

Alongside the sentiment analysis, content analysis of the interview transcripts was also carried out. Fig 12 shows a treemap of the individual nodes with the aggregated nodes represented by the different coloured areas.

Of the aggregated nodes outlined in Fig 12, *Talking & social contact* was one of the most common identified, along with nodes involving support, isolation and recognising good work. Herein, quotes are presented that typify the most common aggregated nodes. For each quote, the aggregated node(s) and individual node(s) it aligns with are shown below, along with the participant the quote is from.

Developing relationships and trust with farmers was considered to take time but the longer-term benefit included helping vets to develop self-confidence in their practice.

“The best bits are once you’ve established a good relationship with the client, and you’ve established trust, and your opinion is very valued by the client.”

Participant 8. Aggregated Node: Job-related, Node: Diagnostics and clinical challenges; Aggregated Node: Talking and social contact, Node: Farmers

“Interacting with clients, making an impact on farms. So, the more time I’ve been in a job, I feel more happy building relationships with different farmers and gives me the knowledge that I know my farmers and I know my farms and I don’t feel lost in what I’m doing.”

Participant 14. Aggregated Node: Job-related, Nodes: Diagnostics and clinical challenges, Good outcomes and recognising positives; Aggregated Node: Talking and social contact, Node: Farmers

During the interviews it was mentioned that hearing about the experiences of others, not least from *“some of the big names in farm animal medicine”* was considered to have a positive influence in helping new graduates and younger vets to gain a better perspective:

“I found it very helpful with my mistakes to hear colleagues talk about their mistakes. I’ve been lucky to have some quite candid about their muck-ups.”

Participant 28. Aggregated Node: Job-related, Nodes: Diagnostics and clinical challenges, Dealing with mistakes; Aggregated Node: Talking and social contact, Node: Talking to others in the team / realising others are going through the same.

“I do try and make sure that all the younger vets and students hear my mistakes, if I can put them in a bit of a humorous light … I try to put a bit of a positive spin on it–at least it made a good story in the end, kind of thing. Even if it was a bit of a disaster, ‘it worked out in the end–I’m still here, I’m still a vet, still enjoying my job’. Things happen to everyone! Things do go wrong and that’s just the nature of the job. It’s not always going to go right.”

Participant 28. Aggregated Node: Job-related, Node: Dealing with mistakes; Aggregated Node: Veterinary practice-related, Nodes: Mentoring and interns, Support from management; Aggregated Node: Self-management, Node: Acceptance and moving on; Aggregated Node: Talking and social contact, Nodes: Joking and humour, Talking to others in the team / realising others are going through the same.

The experiences of others, particularly around mistakes were highlighted in other parts of the interviews. From a management perspective, this dialogue around when outcomes were negative, and mistakes were made, was considered to be an important element of professional development and support for staff.

“… when something goes wrong, they need somebody to pick them up and say, ‘yes, you might have made a mistake but just learn from it and try not to repeat it’.”

Participant 2. Aggregated Node: Job-related, Node: Dealing with mistakes; Aggregated Node: Veterinary practice-related, Nodes: Mentoring and interns, Support from management; Aggregated Node: Self-management, Node: Acceptance and moving on; Aggregated Node: Talking and social contact, Node: Talking to others in the team / realising others are going through the same.

“I think it’s really important that they see it’s normal to make mistakes.”

Participant 6. Aggregated Node: Veterinary practice-related, Node: Mentoring and interns; Aggregated Node: Talking and social contact, Node: Talking to others in the team / realising others are going through the same.

One young vet agreed that hearing about the mistakes made by others *“would definitely help”* as it would make them *“feel better about myself”* knowing that they were *“not the only one”*. As well as individual vets trying to be open about their experiences when mistakes are made, some felt there was a responsibility for ‘practices to make that culture’ of openness.

“… it’s all about being supported, particularly in your first practice, a supportive environment where you can own up to mistakes, learn from them, ask for help if you need it and you don’t live in this world where everyone pretends that everything should be perfect.”

Participant 11. Aggregated Node: Veterinary practice-related, Nodes: Mentoring and interns, Support from management; Aggregated Node: Self-management, Node: Acceptance and moving on; Aggregated Node: Talking and social contact, Node: Asking for help and support.

“Failures and mistakes; we missed a pregnancy and coming back we talked through it with the team here. We have a very supportive team that we can go back to and say, ‘this has happened’.”

Participant 19. Aggregated Node: Job-related, Nodes: Diagnostics and clinical challenges, Dealing with mistakes; Aggregated Node: Veterinary practice-related, Nodes: Mentoring and interns, Support from management; Aggregated Node: Talking and social contact, Node: Talking to others in the team / realising others are going through the same.

Perfectionism was stressed by many vets as problematic in that it resulted in an inability of vets to accept their own mistakes and negative outcomes, saying ‘it must be my fault and I’m a terrible vet, and I should leave the profession’. The importance of accepting that ‘everybody makes mistakes’ was highlighted repeatedly in interviews:

"I’ve had to say, hang on a minute, in what world do you think that you reach a point where you stop making mistakes? It’s not whether you make a mistake, it’s how you respond to it that matters. If they can be more forgiving to other people, they can be more forgiving to themselves."

Participant 11. Aggregated Node: Job-related, Node: Dealing with mistakes; Aggregated Node: Self-management, Node: Acceptance and moving on.

"Not to get too hung up on perfection. If it’s good enough, it’s good enough. Farm animal practice is still a rough and ready business and perfection is not always necessary."

Participant 20. Aggregated Node: Job-related, Node: Dealing with mistakes; Aggregated Node: Self-management, Node: Acceptance and moving on.

Alongside farmer relationships, many vets discussed aspects of working as part of a team as providing job satisfaction. Some described the social aspect of ‘team spirit between the vets’ as being very important in counteracting isolation and in providing support:

"The team is the most fundamental thing we’ve got–our biggest support network … Knowing you’re in a team environment where people have got your back helps, shoulders the pressure, and that’s a huge relief."

Participant 15. Aggregated Node: Veterinary practice-related, Nodes: Fun and team building activities, Support from management; Aggregated Node: Talking & social contact, Nodes: Asking for help and support, Community, Talking to others in the team / realising others are going through the same.

"It’s important to have friends in work".

Participant 20. Aggregated Node: Talking & social contact, Node: Role of family and friends.

"Being part of a supportive good team that will step in and help out if required is a foundation of dealing with difficult days, weeks, years. If people get isolated, that’s when things start to get serious, so I think interaction is the key."

Participant 12. Aggregated Node: Job-related, Node: Working alone; Aggregated Node: Veterinary practice-related, Nodes: Fun and team building activities, Support from management; Aggregated Node: Talking & social contact, Nodes: Asking for help and support, Community, Talking to others in the team / realising others are going through the same.

"If my vet team here wasn’t as good as it is, and I was coming into work dreading it more than I already do, and I’d go home and I’d be on my own, I’d be in a really dark place. Loneliness is crippling."

Participant 15. Aggregated Node: Job-related, Node: Working alone; Aggregated Node: Veterinary practice-related, Nodes: Fun and team building activities; Aggregated Node: Talking & social contact, Nodes: Asking for help and support, Community, Talking to others in the team / realising others are going through the same.

One participant expressed their frustration at a management / business approach which restricted opportunities for team working and meant that individuals increasingly worked alone, when the company of a colleague would help to reduce isolation amongst the staff.

“It can be quite a lonely job, but the big problem is that if you run that past the boss he is always going to say, ‘that doesn’t require two vets’. Seems a real big, mental thing at the moment, where it makes more sense for the vets to be sat in the office rather than sending two vets on the same call!”

Participant 15. Aggregated Node: Job-related, Node: Working alone; Aggregated Node: Veterinary practice-related, Nodes: Mentoring and interns, Support from management.

However, some practices were pro-active in building social cohesion amongst staff, for example by arranging activities outside working hours. Indeed, some vets in a management role described informal social contact outside work as a method to support their younger vets to manage their expectations, especially after a negative outcome. There was consensus about the benefit of being able to share experiences with peers who would understand what it was like.

“… as a team we’re trying socialising outside of work; boot camp–early morning exercises that have been really good.”

Participant 19. Aggregated Node: Veterinary practice-related, Node: Fun and team building activities; Aggregated Node: Exercise / sun / sleep, Nodes: Exercise, keeping fit, playing sport; Outside–sunlight.

“… just talking about it with the rest of the team and reassuring yourself that everyone has the same problem on that farm and it’s not just you, and laughing and joking about it.”

Participant 1. Aggregated Node: Talking and social contact, Nodes: Joking and humour, Talking to others in the team / realising others are going through the same.

“… work with my teammates, and that involves having discussions and regular meetings at work so everyone knows where we’re at, where we’re heading and that gives me job satisfaction.”

Participant 14. Aggregated Node: Veterinary practice-related, Nodes: Mentoring and interns, Support from management; Aggregated Node: Talking and social contact, Node: Talking to others in the team / realising others are going through the same.

## 4. Discussion

The quantitative methods of sentiment and RAKE analysis shown above, identified emotional themes of anticipation and trust along with issues around age of vets and support. In contrast, tf-idf highlighted individual themes, such as religion, not present across all responses. The content analysis supported these findings, with participant quotations revealing examples of trust around relationships with farmers and more experienced vets—along with how farm vets, particularly younger ones, can benefit from good support networks.

Typically, datasets of qualitative data “generally encompass multiple research questions” [40] and researchers undertake coding in their initial data-driven exploratory analysis. There follows a series of analytical decisions about which *chunks* of data are used to ‘tell the story’ and draw conclusions. Researchers are involved in a judgemental process [41]. There is a need to delineate boundaries for data reduction. In the past where data reduction has been required in order to analyse large datasets of qualitative data, techniques such as structural coding, co-occurrence (when two or more codes are used for the same text), and cluster analysis of thematic data have been used [40]. More recently, the emergence of computerised text mining has been mooted as an “efficient and cost-effective solution” to analysis of large qualitative datasets [42]. Guetterman et al [43] found that whilst an automated process such as natural language processing (NLP) can be quicker than the more laborious and resource-intensive traditional approach by researchers, some of the nuances in datasets can be missed and recommend use of both, with the computerised approach augmenting traditional qualitative analysis. Andreotta et al [44] created a framework for integrating computational and qualitative text analyses for a large dataset of qualitative data from social media which included consideration of sentiment.

Our study differs in that the data set was collected by telephone interviews which were transcribed and coded as part of qualitative analysis, with the sentiment analysis undertaken separately. However, the outcomes from both methods show very similar results. The bigrams of *"newer vet"*, *"young graduate"* and *"younger graduate"* from the RAKE analysis was supported in those responses identified within the Talking and Social Contact aggregated node. Similarly, the *"second vet"* bigram supports the node of Working alone identified within the Job-related aggregated node. The trust emotion highlighted as the one of the two most common sentiments, as scored by the nrc lexicon, is widely found within the responses of individual participants. Some of the results also show where the sentiment analysis can add to content analysis with tf-idf results shown in Fig 10 presenting a possible individual coping mechanism of religion that was not identified as a node in the qualitative approach.

The quantitative methods described above allowed the transcribed interviews to be processed objectively with both overall and individual themes identified. One necessary step in the process is the identification of the correct set of stop words, those common words that should be removed from the transcripts prior to analysis. This set of stop words will be unique to the individual context and in this case was produced by collaborative discussion amongst the researchers involved who include both quantitative and qualitative researchers, along with a veterinary epidemiologist. This co-design approach was integral to the entire How Farm Vets Cope project and a critical step in allowing the methods to be amalgamated. This part of the work identified the themes of trust, communication and support to be integral to the experience of practicing farm veterinary surgeons, confirming existing results [1–4].

The two most common emotions identified within the nrc sentiment analysis were trust and anticipation. Examining individual terms associated with these emotions, particularly when combined with the content analysis, identified confidence and support focal points. Lack of self-assurance and confidence is likely to be problematic for vets who need to earn the trust of farmers and in situations where there is a need to consolidate veterinary advice with that of other advisors in a farm management team [45], especially for new graduates and individuals who have very little experience of farm animal handling. Stratham and Green [46] highlight lack of confidence as a factor leading to the drift into small animal practice which they describe as being better supported and less tiring.

There is further cross over between the nrc sentiment analysis and the results of the content analysis when we compare individual words. Fig 8 shows that the only two words with a negative afinn sentiment, risk and worry, were associated with emotion of anticipation. This identification of worry supports earlier work observing cycles of worry [4] and the links to the node of acceptance and moving on. Additionally, the word perfect appears in both word clouds shown in Fig 8 and that idea of perfectionism was highlighted in the quotes shown in section 3.3.

The node of farmer relationships identified as part of the qualitative analysis was not as clearly shown in the quantitative approach. For vets who report finding communication with farmers both challenging and frustrating [47] the process of building trust with farmers is likely to be more problematic. Analysis of role-play interactions of cattle veterinarians revealed a directive style of communication which was paternalistic and not relationship-centred [48]. As different styles of communication are required for different types of farmers [49], vets are required to overcome some hurdles in order to develop trusting relationships.

The relationships vets have with their clients, and the stress that these can cause, were highlighted in several of the articles reviewed by Pohl et al [20]. Amongst these was also a difference depending on the experience of the vet [50,51]. This theme of experience was clearly identified within both the sentiment and content analyses. Several of the participants who self-identified as being more experienced were aware of the support that they could provide to younger vets. Similarly, those who acknowledged they were less experienced sought the support of senior vets and noticed when that support wasn’t there.

The age or experience of a vet should also be taken into consideration when managing expectations in order to be more accepting of self when mistakes are made; it was the experience of older vets and peers which were suggested to have most potency. This focus on experience is highlighted by the results of the RAKE analysis where *“young/younger vet”* and *“new vet”* are identified, and the quotes detailed above from those participants who self-identified as younger. The experience of newly qualified vets being confronted by a sceptical farmer is described as ‘shell shock’ [52]. Previous studies have shown that younger vets and those who are unmarried or without partners report higher stress levels [20,53]. Newly graduated vets face a steep learning curve during which their confidence is put to the test, not least by the need to sound and appear credible in the eyes of farmer clients. As one interviewee noted, the time in a vet’s career at which their confidence may be lowest–just after qualification–is also that time at which they face a significant test of their professional clinical capabilities and their ability to instil confidence in others. As time passes, relationships with clients gain strength, and the ability to manage mistakes improves, so too will clinical skills improve and there may be less often a requirement for mistakes to be managed in the first place. Openness about mistakes is likely to be less uncomfortable amongst colleagues with whom there is a social bond. Practices which encourage and facilitate the time and space for social bonds to be strengthened may benefit from improved stability in their team and wellbeing in their employees [54]. Thus, in relation to both clients and colleagues, the ability to develop and maintain trusting relationships is likely to be key to a vet’s resilience in practice.

As the How Farm Vets Cope project was designed to assist all farm vets, specific information about how long participants had been working or whether they were managers/self-employed was not requested. Within the interviews, many participants provided information on these topics and it was of interest that those who might be classified as having worked for longer have had a greater number of negative experiences but in turn have developed additional coping mechanisms. It is also plausible that those who develop coping mechanisms for the specific challenges faced by a livestock vet might remain working in this field for longer.

With younger or less experienced vets recognised as benefiting from further support and signposting, this confirms the suggestions of Brscic et. al. [19] that preparing veterinary students for the experiences highlighted here will be crucial for their development.

There are several areas where the methodology used in the study could possibly be improved. The interviews were conducted by two members of the study team, with different qualifications and differing levels of experience. Using semi-structured interviews can allow for personal bias, including unconscious biases, to influence the way the interviewer interacts with their participants. Utilising a fixed set of interview questions may remove bias from the interviewer, but it removes the option to gather a wider range of information and in this case, these were crucial to the wider aims of the How Farm Vets Cope project.

Within the quantitative methods, decisions were made around not adding the terms *"good"*, *"vet"*, *"surgeon"* and *"practice"* to the list of stop words. Similarly, the text analysed did not undergo lemmatization, reducing words to their root word, which would have removed the dual occurrence of *"young vet"* and *"younger vet"* in the RAKE analysis. Such decisions will have an impact on the results, particularly when more automated methods are being utilised [55,56].

Triangulation of data as a result of combining quantitative methods with qualitative content analysis and individual quotes allows not only for identification of more themes/topics/nodes overall but also for a more nuanced discussion than is possible with only the quantitative approach. An example of this is the bigram of *"WhatsApp group"* which was identified by both RAKE and tf-idf for one participant. Upon investigation, the bigram was used by four participants, with each participant identifying a *"WhatsApp group"* that was set up to help within a practice, either from a personal or clinical point of view. However, the reaction to these groups within the individual practices differed. If follow up interviews had been planned as part of the project, this could be studied further.

The 30 interviews processed are a small enough collection that they could be processed solely utilising qualitative methods, but the authors consider the combined approach has allowed for in depth analysis and would be far easier to scale up to a larger set of interviews, whilst–through the use of quantitative methods such as RAKE and tf-idf in particular–retaining the capacity to identify results that may not be common across a cohort, but which are significant at the individual level.

## Supporting information

S1 Checklist In our supporting information we have provided a completed Consolidated criteria for reporting qualitative research (COREQ) checklist for the above work in the supplementary file COREQ_Checklist. (PDF)
