# Supplementary material for: Combining sentiment analysis and text mining with content analysis of farm vet interviews on mental wellbeing in livestock practice
Source: PLoS One. 2024 May 22;19(5):e0304090. doi: 10.1371/journal.pone.0304090 (PMC11111023; doi:10.1371/journal.pone.0304090)
Supplement: S1 Checklist — (PDF) [file pone.0304090.s001.pdf]

Combining sentiment analysis and text mining with content analysis of farm vet interviews on mental wellbeing in livestock practice: Supplementary Information- COREQ Checklist

Andrew J. Duncan[1, 2*], Madeleine K. Henry[1], Kate Lamont[1]

[1]Centre for Epidemiology and Planetary Health, Department of Veterinary and Animal Science, Northern Faculty, Scotland's Rural College (SRUC), Inverness, UK

[2]UHI Inverness, University of the Highlands and Islands, 1 Inverness Campus, Inverness, IV2 5NA, UK
*****Corresponding Author: andrew.duncan.ic@uhi.ac.uk

COREQ Checklist

In the following tables (SI Tab1 – 3), the checklist for the three domains (Research team and reflexivity, study design and analysis and findings) are shown.

SI Tab 1: The COREQ checklist for domain 1 - Research team and Reflexivity.

**Domain 1: Research team and reflexivity**		
		Personal Characteristics
1.	Interviewer/facilitator	Which author/s conducted the interview or focus group?<br>KL was the principal interviewer; MKH also conducted some of the interviews.
2.	Credentials	What were the researcher's credentials? *E.g. PhD, MD*<br>KL – PhD; MKH - MSc
3.	Occupation	What was their occupation at the time of the study?<br>Both KL and MH were employed as research staff within the Epidemiology Research Unit at SRUC, now the Centre for Epidemiology and Planetary Health. KL working predominantly in the field of social science and MKH in the field of veterinary epidemiology.
4.	Gender	Was the researcher male or female?<br>Both researchers who conducted the interviews were female.
5.	Experience and training	What experience or training did the researcher have?<br>KL had extensive experience interviewing participants in social science projects. MKH had experience as a member of the veterinary profession interacting with fellow members both professionally and personally.
		Relationship with participants
6.	Relationship established	Was a relationship established prior to study commencement?<br>No.

7.	Participant knowledge of the interviewer	What did the participants know about the researcher? e*.g. personal goals, reasons for doing the research*<br><br>KL made initial contact with all participants when they notified her of their interest in being involved in the project. All participants were provided with an information sheet about the aims of the project. Each interviewer introduced themselves and their background at the beginning of the interview.
8.	Interviewer characteristics	What characteristics were reported about the interviewer/facilitator? e.g. *Bias, assumptions, reasons and interests in the research topic*<br><br>Both interviewers reflected on their practice throughout the project and used standardised materials/prompts for all interviews. Although the analysis was primarily carried out by KL, it was reviewed by MKH. This process helped identify potential bias and challenged assumptions.

SI Tab 2: The COREQ checklist for domain 2 - Study design

**Domain 2: Study design**		
	Theoretical framework	
9.	Methodological orientation and Theory	What methodological orientation was stated to underpin the study? *e.g. grounded theory, discourse analysis, ethnography, phenomenology, content analysis*<br>Grounded theory.
	Participant selection	
10.	Sampling	How were participants selected? *e.g. purposive, convenience, consecutive, snowball*<br>Purposive.
11.	Method of approach	How were participants approached? e.*g. face-to-face, telephone, mail, email*<br>Recruitment was done via the use of direct messages on Twitter, email and phone contact with veterinary surgeons who previously indicated a willingness to participate, and through 'sign up' fliers at the British Cattle Veterinary Association Congress in October 2019. In addition, some veterinary practices and groups of farm veterinary practices shared information about the project to their staff.<br>Vets who expressed an interest in taking part were sent a participant information sheet and consent form by email. Two follow up emails were sent if no response was received. Participants were recruited in the period between September 20th, 2019 and February 28th, 2020.
12.	Sample size	How many participants were in the study?<br>A total of 85 individual expressions of interest were received and information about the project provided to each. 32 interviews were consented to, 31 were successfully arranged and 30 of these were transcribed for analysis.
13.	Non-participation	How many people refused to participate or dropped out? Reasons?

		We were unable to arrange an interview with one participant who had already consented and this wasn't followed up at a later date due to data saturation considerations (please see below).
	Setting	
14.	Setting of data collection	Where was the data collected? e.*g. home, clinic, workplace*<br><br>The interviews were conducted mostly in the workplace, with some out of office hours interviews arranged at the convenience of the individual interviewees.
15.	Presence of non-participants	Was anyone else present besides the participants and researchers?<br><br>The researcher conducted the phone interviews in a private room; we cannot be sure about whether or not there were other people present with the participant.
16.	Description of sample	What are the important characteristics of the sample? *e.g. demographic data, date*<br><br>Participants were required to have experience of working as a farm vet in the UK. No other demographic characteristics were collected but some were volunteered as part of the interview.
	Data collection	
17.	Interview guide	Were questions, prompts, guides provided by the authors? Was it pilot tested?<br><br>The interviews were semi-structured, with three broad topic areas for discussion but the flexibility to allow the conversation to move onto related matters if the participant led it in that direction. An initial informal interview was undertaken to pilot the questions/prompts.
18.	Repeat interviews	Were repeat interviews carried out? If yes, how many?<br><br>No repeat interviews were carried out.
19.	Audio/visual recording	Did the research use audio or visual recording to collect the data?<br><br>Yes, audio recording was used to allow phone conversations to be accessed again later by the researcher who conducted the interview and the team member responsible for transcription.
20.	Field notes	Were field notes made during and/or after the interview or focus group?<br><br>Yes, the researchers made notes while speaking on the phone with the participants.

21.	Duration	What was the duration of the interviews or focus group?<br><br>Target duration was 30 minutes, although some interviews were shorter and others much longer. The length of time was governed by how much the participant wanted to say about each of the topic areas covered.
22.	Data saturation	Was data saturation discussed?<br><br>Yes. After completion of the 31 interviews, researchers examined the data collected and made the collective decision that further interviews did not need to be conducted because data saturation had been reached.
23.	Transcripts returned	Were transcripts returned to participants for comment and/or correction?<br><br>An interview transcript was returned to one participant due to audio quality during the interview itself.

SI Tab 3: The COREQ checklist for domain 3 - Analysis and findings.

Domain 3: Analysis and findings		
		Data analysis
24.	Number of data coders	How many data coders coded the data?<br>KL coded the data and the results were reviewed by MHK.
25.	Description of the coding tree	Did authors provide a description of the coding tree?<br>A coding tree was available to the research team through NVivo.
26.	Derivation of themes	Were themes identified in advance or derived from the data?<br>Themes or rather nodes were derived from the data. Nodes were further grouped into aggregated nodes for comparison with quantitative results.
27.	Software	What software, if applicable, was used to manage the data?<br>NVivo was used to manage the data.
28.	Participant checking	Did participants provide feedback on the findings?<br>Feedback was provided during a focus group workshop.
		Reporting
29.	Quotations presented	Were participant quotations presented to illustrate the themes / findings? Was each quotation identified? e.*g. participant number*<br>Yes, quotations were presented with aggregated node/node and participant (via anonymous numerical ID) identified.
30.	Data and findings consistent	Was there consistency between the data presented and the findings?<br>Yes.

31.	Clarity of major themes	Were major themes clearly presented in the findings?<br>Both the aggregated nodes are clearly identified in Fig 12 of the publication.
32.	Clarity of minor themes	Is there a description of diverse cases or discussion of minor themes?<br>Yes. Individual nodes are identified in Fig 12 of the publication and included in the discussion.
